# Factors influencing objective voice profile and speech intelligibility of patients after oral oncological treatment: a prospective cohort study

**DOI:** 10.1007/s00520-025-09793-z

**Published:** 2025-07-31

**Authors:** Jenthe Thienpondt, Kris Demuynck, Matthias A. W. Merkx, Caroline M. Speksnijder

**Affiliations:** 1 IDLab, Department of Electronics and Information Systems, Ghent University - Imec, Ghent, Belgium; 2 Department of Research and Development, Netherlands Comprehensive Cancer Organisation (Integraal Kankercentrum Nederland, IKNL), Utrecht, The Netherlands; 3 IQ Healthcare, Radboud University Medical Center, Nijmegen, The Netherlands; 4 Department of Oral and Maxillofacial Surgery, Radboud University Medical Center, Nijmegen, The Netherlands; 5 Department of Oral and Maxillofacial Surgery and Special Dental Care, University Medical Center Utrecht, Utrecht University, P.O. Box 85.500, 3508 GA Utrecht, The Netherlands; 6 Department of Head and Neck Surgical Oncology, University Medical Center Utrecht, Utrecht University, Utrecht, The Netherlands

**Keywords:** Oral cancer, Voice profile, Speech intelligibility, Speaker embeddings, Automatic speech recognition, Linear mixed-effects analysis

## Abstract

**Purpose:**

While objective assessment of speech intelligibility after oral oncological treatment has proven reliable, no such method currently exists for assessing changes in perceptual voice profile. In this study, we introduce a novel objective method to assess changes in perceptual voice profile after oral oncological treatment. Subsequently, we identify demographic and clinical factors associated with objective assessments of voice profile and intelligibility from the pre-treatment stage up to 12 months after treatment.

**Methods:**

Speech samples were collected from 140 patients pre-treatment (baseline) and 1, 6, and 12 months post-treatment. Neural speaker embeddings were used to measure changes in voice profile relative to the pre-treatment stage while an automatic speech recognition system determined speech intelligibility. A linear mixed-effects model was used to associate demographic and clinical factors with both measurements over time.

**Results:**

Voice profile and speech intelligibility of patients were significantly impacted 1, 6, and 12 months after treatment compared to the baseline measurements. Increased age and tobacco usage were associated with worse intelligibility before treatment. Advanced tumor stage, bone flap reconstruction, local flap reconstruction, and their interaction with the timing of assessment were associated with both voice profile and intelligibility. Radiotherapy was associated with changes in voice profile 6 and 12 months after treatment but did not interact significantly with intelligibility.

**Conclusion:**

Voice profile and speech intelligibility are significantly affected after oral oncological treatment. The severity is influenced by age, tobacco usage, tumor stage, and surgical reconstruction type. Radiotherapy seems to change voice profile rather than reducing intelligibility.

**Supplementary Information:**

The online version contains supplementary material available at 10.1007/s00520-025-09793-z.

## Introduction

Oral cancer is a potentially life-threatening type of cancer that can arise in different areas of the mouth, primarily stemming from tissues in the oral cavity [1]. Common treatment options involve surgery, radiation, and chemotherapy, either individually or combined, depending on the cancer stage and location. Oral oncological treatment inevitably affects the morphological structure of the vocal tract, often resulting in impaired oral functionality [2]. Inhibited oral functionality can encompass a wide range of interconnected problems related to deglutition, mastication, nutrition, social interaction, and speech [3–5].

Impaired speech outcomes after oral oncological treatment are related to diminished quality of life of patients [5, 6], indicating the importance of identifying contributing demographic and clinical factors. Previous studies have suggested that several clinical factors can influence speech outcomes, including tumor site, surgical reconstruction type, treatment modality, and tumor stage [7, 8], in addition to demographic factors such as age, alcohol, and tobacco usage [9, 10].

Diminished speech-related outcomes (i.e., outcomes related to functional human communication) after oral oncological treatment can manifest as reduced articulation, intelligibility, and verbal fluency [11, 12]. Concurrently, patients can also show changes in attributes related to voice profile, including altered pitch, hoarseness, and breathiness [13, 14]. In this paper, we refer to voice profile as the collection of identifying perceptual characteristics of each person’s voice, all contributing towards the ability of naive listeners to discriminate between speakers based on voice samples as employed in [15].

Most studies rely on subjective perceptual methods to assess speech outcomes after oral oncological treatment, usually by patient questionnaire or expert evaluation [13, 14]. Perceptual assessment by speech therapists is currently considered the best method to evaluate speech outcomes. However, this procedure is error-prone, resource-intensive, and time-consuming [16]. Consequently, there is a growing need for reliable, objective, and automatic evaluation of speech outcomes after oral cancer treatment.

Previous studies have attempted to objectively assess speech outcomes after oral oncological treatment [13, 14, 17]. Most promising has been the employment of automatic speech recognition (ASR) models as an objective method to measure speech intelligibility. In this paper, we refer to speech intelligibility as the acoustic–phonetic decoding of an utterance as defined in [18]. ASR systems apply acoustic and language models to decode spoken input into the corresponding text [19]. Research has repeatedly shown that ASR word error rate (WER) correlates strongly negatively (*r* =  *− *0*.*93*, P* < *0.0*1) [20] with expert evaluation of speech intelligibility in both healthy and pathological speakers [21, 22]. Primary limitations of ASR-based techniques lie in the requirement of a reference transcription, sensitivity to recording conditions, and its language dependency. In addition, ASR-based intelligibility measurements do not provide information regarding changes in voice profile. In this regard, several studies evaluate specific acoustic parameters of the voice after oral oncological treatment such as fundamental frequency, jitter, and shimmer [23, 24]. Although these parameters all contribute towards the identifying perceptual characteristics of an individual’s voice profile, a single encompassing objective metric to evaluate changes in voice profile following oral oncological treatment is missing.

To address this concern, we argue that recent advancements in neural speaker embeddings provide a compelling perspective. Speaker embeddings are fixed-size numerical representations that capture the identifying characteristics of a person’s voice based on short speech utterances. The similarity of embeddings reflects how similar the speakers’ voices are, usually quantified by cosine similarity or related metrics [25]. Previous research denoted a strong correlation between voice similarity ratings based on current state-of-the-art speaker embeddings and human perception (*r* = 0*.*82) [15]. Our prior work effectively employed neural speaker embeddings derived from speech samples taken before and after oral oncological treatment of patients to measure changes in voice profile during the treatment trajectory [26]. However, this work was restricted to a preliminary descriptive analysis and a limited number of patients. Therefore, we aim in this prospective study to identify clinical factors associated with objective changes in voice profile in patients following oral oncological treatment. In addition, we employ an ASR model to measure speech intelligibility of patients during their treatment trajectory. This approach allows us to evaluate and compare the effects of clinical factors on objective metrics of both voice profile and speech intelligibility.

## Materials and methods

### Study design and population

Speech samples were collected from 140 patients aged 18 years or older, diagnosed with a primary malignant tumor affecting the oral cavity and undergoing oncological treatment at the University Medical Center Utrecht (UMCU) or Radboud University Medical Center (Radboudumc) between January 2007 and August 2009. Participants were eligible for inclusion if they were treated with a curative intent, either by surgery or by (adjuvant) radiotherapy. Exclusion criteria were inoperable conditions, a prior or concurrent second primary malignancy, impaired cognition or lack of proficiency in Dutch. No oral cancer types were excluded.

The tumor locations of the oral cancers included the codes C00, C02 to C06, and C31 of the WHO International Classification of Diseases Oncology third edition (WHO ICD-0–3) [27]. Maxillary tumors included those on the upper alveolar process, tuber maxillae, palate, and maxillary sinus (C03.0, C05, C31.0). Mandibular tumors included those on the lower alveolar process, the retromolar trigonum, the buccal mucosa, and the lower lip (C00.4, C03.1, C06.0, C06.1, C06.2). Tongue and floor-of-the-mouth tumours included those located on the tongue and the anterior floor of the mouth (C02, C04).

The study protocol obtained approval from the Ethics Committee of the UMCU and Radboudumc (NL.12006.041.06) in accordance with the Declaration of Helsinki. Sixty healthy controls matched for age and gender were also included, whose details were published previously [28]. All participants provided written informed consent.

### Data collection

Patient speech samples were acquired at the following measurement moments: within 4 weeks before oncological treatment (M0), 4 to 6 weeks after surgery or (adjuvant) radiotherapy (M1), and 6 (M6) and 12 (M12) months post-treatment. Samples were only collected once for the healthy individuals. During each sampling session, speakers read two Dutch and phonetically diverse texts, allowing for reliable speech analysis. The first text (*text1*, Appendix A) is a speech test proposed in Dysarthria and Apraxia of Speech [29] while the second text (*text2*, Appendix B) is a similar test designed by speech therapists for additional data collection. The recording equipment consisted of a Logitech USB Desktop Microphone (Logitech® A-0186A, Newark, CA, USA) and conditions were standardized across all sessions with the microphone placed in front of each subject with a 30-cm mouth-to-microphone distance.

### Clinical and demographic factors

The clinical factors included in this analysis were as follows: surgical reconstruction type (primary closure, local flap, free flap, or bone flap), treatment modality (radiotherapy, surgery, or surgery with adjuvant radiotherapy), tumor site (maxilla, mandible, and tongue/floor of mouth (TFM)), and tumor stage (T1 to T4 of TNM [30]). A small number of patients (*n* = 6) had received chemotherapy. However, this was not included in our analysis to avoid too sparsely populated classes in the treatment modality category. In addition, the following demographic factors were collected at M0: sex (male or female), age (continuous), current alcohol consumption exceeding 1 unit on average each day (yes or no), and current daily tobacco usage (yes or no).

### Voice profile

To assess the evolution of voice profile in patients undergoing oral oncological treatment, we employ our state-of-the-art ECAPA2 speaker embedding model (huggingface.co/Jenthe/ECAPA2) to extract embeddings from all collected speech samples [31]. We use the publicly available version of this model to enable comparison and reproducibility with future research. Details about acoustic feature pre-processing, model architecture, and training procedure are available in the accompanying paper [31]. ECAPA2 speaker embeddings are trained to maximize the cosine similarity between embeddings of the same speaker, providing a comparative, encompassing, and objective metric of voice profile similarity between speech samples.

**Fig. 1:**
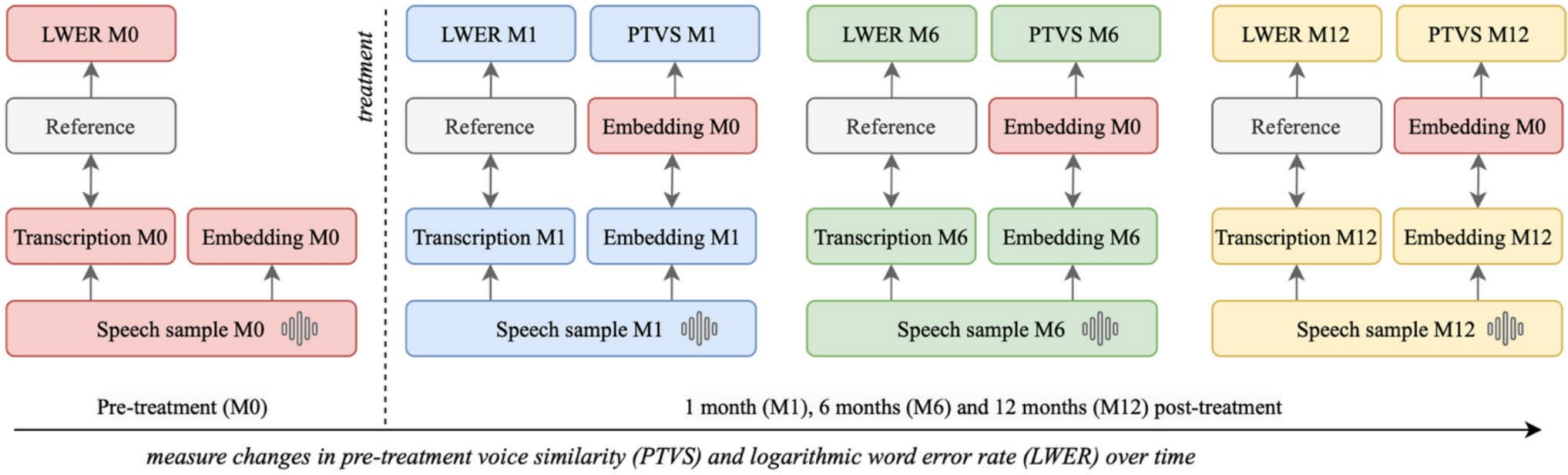
Overview of methodology to measure evolution of voice profile (PTVS) and speech intelligibility (LWER) of a patient

We use this metric to model the evolution of voice profile from patients after treatment, relative to the pre-treatment measurement moment. The pre-treatment *text1* embedding at M0 represents the baseline. Subsequently, similarity scores are computed speaker-wise with the *text2* embeddings at all measurement moments in the treatment trajectory (Fig. 1). Additionally, similarities between the *text1* and *text2* embeddings of healthy speakers are calculated as a comparison.

To adhere to model assumptions in the statistical analyses, we employ the logarithmic angle between embeddings instead of the cosine similarity as our comparative metric. To improve interpretability, we map the raw similarity scores to proper log-likelihood-ratios (LLRs) by training a linear regression based calibration system as described in [32] using the VoxCeleb1 [33] test set. Our final metric indicates the pre-treatment voice similarity (PTVS) and is given by:

$$\mathrm{PTVS}\;=\alpha\;\log\;(\cos^{-1}\;(t_1,\;t_2))\;+\beta$$

with *t*_1_ and *t*_2_ being the *text1* and *text2* speaker embeddings, respectively. *α* indicates the trained calibration scalar and *β* the corresponding bias. Specifically, the PTVS metric indicates the loglikelihood ratio between the hypothesis that *t*_1_ and *t*_2_ originate from the same speaker and the null hypothesis that they were produced by different speakers. In this context, a lower PTVS score indicates a greater shift in overall voice profile relative to the pre-treatment state of the patient. Concretely, lower PTVS scores are associated with a lower chance of listeners (e.g., family members) rating pre- and post-treatment utterances of patients as originating from the same speaker [15].

### Speech intelligibility

To assess speech intelligibility, we employ an ASR model based on the XSL-R architecture proposed in [34]. The XSL-R model is pre-trained using a self-supervised approach on cross-lingual and unlabeled adult speech corpora. The aim for the model is to learn contextual representations that accurately reflect both the acoustic and linguistic aspects of the input utterance. More details about the pre-training procedure can be found in the accompanying paper [34]. Afterwards, the model is fine-tuned by addition of a linear layer to project the context representations to the vocabulary of the ASR task. We fine-tune the pre-trained model on the Dutch subset of the publicly available Mozilla Common Voice dataset [35]. Subsequently, we extract transcriptions from the *text1* and *text2* speech samples of each patient and calculate the logarithmic word error rate (LWER) between the prescribed reading text and the ASR transcription output (Fig. 1) to adhere to model assumptions in the statistical analyses.

### Statistical analyses

The relationship between the healthy control group and patients for PTVS and LWER at the descriptive level was determined using independent *t*-tests or Mann–Whitney *U* tests, as appropriate. A statistical analysis was performed using linear mixed-effects models (LMMs) with the dependent outcome variables being the PTVS and LWER scores to examine temporal changes and assess the impact of demographic and clinical factors on changes in voice profile and speech intelligibility, respectively. Random slopes and intercepts were included to account for within-patient correlations and initial patient conditions, respectively. The LMM included as fixed-effects sex, age, alcohol usage, tobacco usage, tumor site, surgical reconstruction type, treatment modality, and tumor stage, as well as their two-way interaction with the timing of assessment.

The model was refined using step-wise backward elimination, discarding non-significant factors with *P*-values ≥ *0.1*0, beginning with the interaction terms. Throughout the process, a hierarchical structure was preserved, ensuring that if an interaction term remained, its corresponding main effects were also included. The variance components and fixed effects parameters of the LMM were determined using restricted maximum likelihood (REML). Model assumptions were verified by a residual analysis and *P*-values ≤ 0.05 were considered statistically significant. The statistical analysis was performed using R version 4.4.0 (R Foundation for Statistical Computing, Vienna, Austria).

## Results

### Patient characteristics

**Fig. 2:**
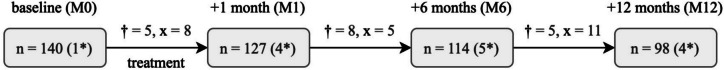
Flowchart of patient participation during the study. The number of missed measurement moments is indicated by *. The number of patients that passed away or stopped participation are given by † and x, respectively

Baseline characteristics of patients enrolled in this study are described in Table 1. After applying the exclusion criteria, 140 patients were enrolled in this study. Among these individuals, all were treated with curative intent, 58 were treated with surgery, 20 with radiotherapy, and 62 received surgery followed by radiotherapy. In total, 139 patients recorded speech samples at M0, 123 at M1, and 109 at M6 and 94 at M12. Over the course of 12 months, 18 patients died and 24 ceased participation (Fig. 2).

**Table 1:** Demographic and clinical characteristics of study patient (_*n*_ = 140) and healthy (_*n*_ = 60) groups

Patient participants	n*	%*
Age (mean _±_ SD)	65.5	12.8
Sex		
* Male*	77	55.0
* Female*	63	45.0
Tobacco usage		
* Yes*	88	62.9
* No*	52	37.1
Alcohol usage		
* Yes*	94	67.1
* No*	46	32.9
Tumor site		
* Maxilla*	33	23.6
* Mandible*	52	37.1
* TFM*	55	39.3
Tumor stage		
* T1*	45	32.1
* T2*	40	28.6
* T3*	10	7.1
* T4*	45	32.1
Treatment modality		
* Surgery*	58	41.4
* Radiotherapy*	20	14.3
* Surgery* + *radiotherapy*	62	44.3
Reconstruction type		
* Primary closure*	54	45.0
* Local flap*	4	3.3
* Free flap*	41	34.2
* Bone flap*	21	17.5
Healthy participants	n*	%*
Age (mean _±_ SD)	60.3	6.9
Sex		
* Male*	31	51.6
* Female*	29	48.4

*Unless noted otherwise

*SD* standard deviation

The mean LWER of the healthy control group (− 1.38 ± 0.42) is overall significantly lower compared to the patients at M0 (− 1.16 ± 0.41, *P* < *0.0*01), M1 (− 0.98 ± 0.48, *P* < *0.0*01), M6 (− 1.08 ± 0.49, *P* < *0.0*01), and M12 (− 1.15 ± 0.48, *P* = *0.0*02), indicating reduced speech intelligibility in patients. PTVS scores of patients were significantly lower than the healthy control group (4.87 ± 0.76) after treatment at M1 (2.54 ± 1.08, *P* < *0.0*01), M6 (2.57 ± 1.02, *P* < *0.0*01), and M12 (2.67 ± 0.92, *P* < *0.0*01), indicating a significant change in voice profile post-treatment.

### Linear mixed-effects analysis

**Fig. 3:**
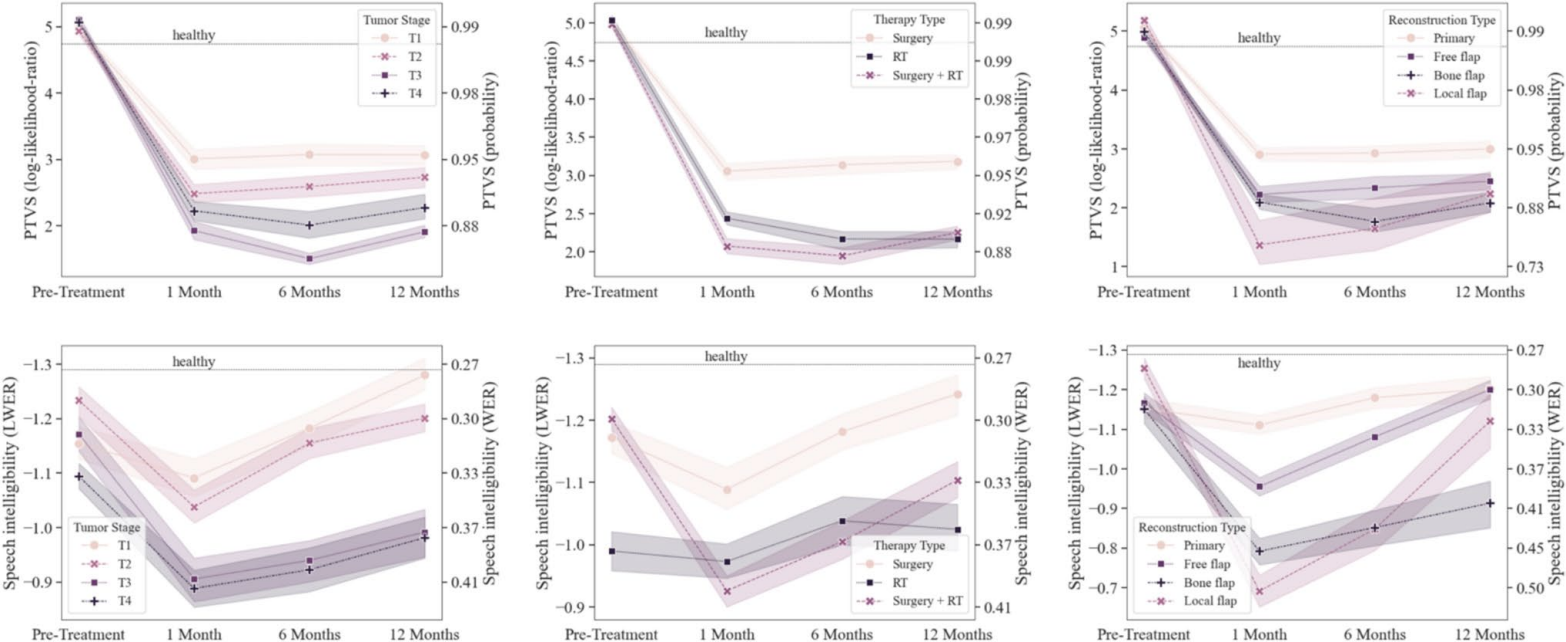
Mean LMM patient outcomes for PTVS (top) and LWER (bottom), grouped by tumor stage, therapy type and reconstruction type. Colored areas indicate corresponding standard deviation. The dotted line depicts the mean PTVS and LWER scores of the healthy control group

The results of the LMM analysis of voice profile and speech intelligibility are shown in Tables 2 and 3, respectively. Corresponding PTVS and LWER estimation formulas are given in Appendix C. Speech intelligibility was negatively associated with age (+ 0.012 LWER/year, *P* < *0.0*01) and tobacco usage (+ 0.193 LWER, *P* < *0.0*1), although both were timing independent. Additionally, changes in speech intelligibility and voice profile were associated with tumor stage, reconstruction type, therapy type, and their interaction with the timing of assessment. Plots depicting the mean LMM patient outcome and standard deviation subdivided by tumor stage, reconstruction type, and therapy type are given in Fig. 3.

**Table 2:** Linear mixed-effects model results for voice profile (PTVS)

	Independent		Interaction with measurement moment					
			*1 month*		*6 months*		*12 months*	
	Estimate (95% CI)	*P*-value	Estimate (95% CI)	*P*-value	Estimate (95% CI)	*P*-value	Estimate (95% CI)	*P*-value
Intercept	5.23 (4.6, 5.85)	< *.***001**						
Stage								
* Baseline*				*reference*				
* 1 month*	− 1.66 (− 1.96, − 1.36)	< *.***001**						
* 6 months*	− 1.64 (− 1.94, − 1.34)	< *.***001**						
* 12 months*	− 1.65 (− 1.96, − 1.33)	< **.001**						
T of TNM								
* 1*				*reference*				
* 2*	− 0.01 (− 0.37, 0.36)	*.*97	− 0.32 (− 0.73, 0.1)	.13	− 0.26 (− 0.68, 0.16)	*.*22	− 0.17 (− 0.61, 0.27)	*.*44
* 3*	0.22 (− 0.38, 0.81)	*.*48	− 0.85 (− 1.5, − 0.19)	*.***01**	− 1.21 (− 1.89, − 0.53)	< *.***001**	− 0.85 (− 1.55, − 0.14)	*.***02**
* 4*	0.18 (− 0.25, 0.61)	*.*41	− 0.41 (− 0.91, 0.09)	.10	− 0.39 (− 0.94, 0.15)	*.*16	− 0.27 (− 0.82, 0.28)	*.*34
Reconstruction								
* Primary*				*reference*				
* Local flap*	0.09 (− 0.75, 0.93)	*.*83	− 1.51 (− 2.43, − 0.6)	*.***001**	− 1.11 (− 2.04, − 0.17)	*.***02**	− 0.74 (− 1.68, 0.2)	*.*12
* Free flap*	− 0.16 (− 0.51, 0.2)	.38	− 0.27 (− 0.67, 0.13)	.19	−0.01 (− 0.43, 0.41)	.95	− 0.13 (− 0.58, 0.32)	.56
* Bone flap*	− 0.15 (− 0.62, 0.32)	.52	− 0.43 (− 0.96, 0.11)	.12	− 0.61 (− 1.22, − 0.0)	**.05**	− 0.57 (− 1.2, 0.05)	.07
Therapy								
* Surgery*				*reference*				
* RT*	− 0.07 (− 0.56, 0.42)	*.*78	− 0.53 (− 1.11, 0.05)	.07	− 0.82 (− 1.46, − 0.18)	*.***01**	− 0.95 (− 1.61, − 0.29)	*.***005**
* Surgery* + *RT*	− 0.05 (− 0.39, 0.29)	*.*79	− 0.58 (− 0.96, − 0.19)	*.***003**	− 0.79 (− 1.19, − 0.38)	< *.***001**	− 0.57 (− 1.0, − 0.14)	*.***009**

*CI* confidence interval, *RT* radiotherapy, **bold**: *P* ≤.05

**Table 3:** Linear mixed-effects model results for speech intelligibility (LWER)

	Independent		Interaction with measurement moment					
			*1 month*		*6 months*		*12 months*	
	Estimate (95% CI)	*P*-value	Estimate (95% CI)	*P*-value	Estimate (95% CI)	*P*-value	Estimate (95% CI)	*P*-value
Intercept	− 1.97 (− 2.36, − 1.58)							
Age	0.01 (0.01, 0.02)	< *.***001**						
Stage								
* No*				*reference*				
* Yes*	0.19 (0.05, 0.34)	*.***01**						
Stage								
* Pre-treatment*				*reference*				
* 1 month*	− 0.01 (− 0.11, 0.08)	*.*76						
* 6 months*	− 0.07 (− 0.16, 0.03)	*.*17						
* 12 months*	− 0.12 (− 0.22, − 0.01)	*.***02**						
T of TNM								
* 1*				*reference*				
2	− 0.08 (− 0.27, 0.12)	.44	0.12 (− 0.02, 0.25)	.09	0.09 (− 0.05, 0.23)	.19	0.18 (0.03, 0.32)	.**01**
* 3*	− 0.06 (− 0.38, 0.26)	*.*72	0.12 (− 0.1, 0.33)	.28	0.18 (− 0.04, 0.4)	*.*10	0.24 (0.01, 0.47)	*.***04**
* 4*	− 0.04 (− 0.27, 0.19)	*.*74	0.04 (− 0.12, 0.2)	.64	0.09 (− 0.09, 0.27)	*.*34	0.15 (− 0.03, 0.33)	*.*10
Reconstruction								
* Primary*				*reference*				
* Local flap*	− 0.19 (− 0.64, 0.26)	*.*41	0.48 (0.19, 0.78)	*.***001**	0.34 (0.03, 0.64)	*.***03**	0.08 (− 0.22, 0.39)	*.*59
* Free flap*	0.02 (− 0.17, 0.21)	*.*86	0.09 (− 0.04, 0.22)	.17	− 0.01 (− 0.15, 0.13)	*.*89	− 0.1 (− 0.25, 0.05)	*.*18
* Bone flap*	0.02 (− 0.23, 0.28)	*.*85	0.28 (0.1, 0.45)	*.***002**	0.21 (0.01, 0.41)	*.***04**	0.16 (− 0.05, 0.37)	*.*13
Therapy								
* Surgery*				*reference*				
* RT*	0.12 (− 0.14, 0.39)	*.*37	0.01 (− 0.18, 0.2)	*.*89	− 0.02 (− 0.24, 0.19)	*.*82	0.02 (− 0.2, 0.24)	*.*85
* Surgery* + *RT*	− 0.02 (− 0.21, 0.16)	*.*80	0.1 (− 0.03, 0.23)	*.*12	0.13 (− 0.01, 0.26)	*.*06	0.12 (− 0.02, 0.26)	*.*08

*CI* confidence interval, *RT* radiotherapy; **bold**: *P* ≤.05

### Tumor stage

Patients with T3 tumors had a significant and severe degradation in their PTVS score in comparison to patients with T1 tumors during all post-treatment measurement moments (*P* < *0.0*5). For intelligibility, patients with a T2 or T3 tumor had a significant degradation in LWER 12 months after treatment compared to the T1 group (*P* < *0.0*5).

### Surgical reconstruction type

Patients undergoing local flap surgery had a significant degradation in both PTVS and LWER at the M1 and M6 measurement moments in comparison to the reference group treated with primary closure (*P* < *0.0*5). In addition, bone flap surgery resulted in a significant reduction in LWER compared to primary closure at M1 (*P* < *0.0*01) and M6 for both PTVS and LWER (*P* < *0.0*5).

### Therapy type

We observe a significant impact on PTVS with patients undergoing radiotherapy 6 and 12 months after treatment, irrespective of preceding surgery, compared to the surgery-only group (*P* < *0.0*1). Patients with adjuvant radiotherapy also exhibited a significant degradation in PTVS (*P* < *0.0*01) at the 1-month measurement moment. No significant interactions were apparent for speech intelligibility.

## Discussion

Similar to previous studies [36, 37], speech intelligibility is overall lower for patients with oral cancer compared to the healthy control group before treatment (*P* < *0.0*01). Increasing age and tobacco usage were independent factors associated with reduced speech intelligibility. The reduction of speech intelligibility in elderly is often reported due to age-related changes in auditory processing, cognitive decline, and deterioration in speech production mechanisms [9, 38]. The interaction between tobacco usage and intelligibility is more difficult to assess, as smoking behavior has many co-dependent variables not included in this study. However, it is suggested that smoking does significantly alter the first four formant frequencies (F1, F2, F3, and F4) [39, 40]. In contrast, no association was found between PTVS and the demographic characteristics in this analysis. In the context of objectively evaluating changes in voice profile, this behavior is favorable, as it ensures robustness against demographic variability without reliance on the statistical model. In addition, an overall significant change in voice profile can be observed at all measurement moments after treatment. Other studies focusing on post-treatment acoustic analysis found changes in spectral properties related to voice profile such as fundamental frequency and harmonics-to-noise ratio [40]. However, it is currently unclear how these acoustic properties relate to perceptual changes in voice profile [41]. As expected, *text1* and *text2* embedding similarity was high at M0, indicating that the speaker embeddings can correctly capture the identity of patients included in this study before treatment.

Changes in voice profile and diminished speech intelligibility were moderately correlated (*r* =  *− *0*.*41*, P* < *0.0*01), denoting a possible mutual dependency on underlying speech measures or the anatomical structure of the vocal tract (Appendix D). Simultaneously, it highlights the complementarity of both metrics in post-treatment speech analysis. Other studies comparing acoustic properties affecting voice profile and intelligibility post-treatment found significant correlations between shifts in certain formant frequencies and perceived intelligibility [42, 43].

Several clinical factors interacted significantly with the timing of assessment over the 1-year course after treatment. Our results suggest that tumor stage significantly influences both speech intelligibility and voice profile. A general trend towards recovery to pre-treatment speech intelligibility can be observed for all tumor stages. However, patients with T2 and T3 tumors exhibited significantly lower intelligibility 1-year post-treatment compared to the T1 group, signifying the negative impact of higher tumor stages on speech intelligibility. This corroborates many previous studies that found a correlation between speech intelligibility and tumor stage [12, 36, 44]. A few studies also evaluated the relationship between tumor size and spectral properties. For example, an objective acoustic–phonetic speech study in patients treated for oral or oropharyngeal cancer found that patients with smaller tumors exhibited a longer duration of air pressure release in plosives in comparison to patients with larger tumors [42] while a longitudinal observational study based on a questionnaire and acoustic analysis study in patients with tongue squamous cell carcinoma noticed a significant decrease in F2 of the vowel/i/among patients with higher T stages [7], both possibly contributing towards changes in speech outcomes. Similar to intelligibility, tumor stage seemed to impact voice profile. This was mostly notable in patients with T3 tumors, who exhibited a significant and severe change in voice profile over the 1-year treatment course compared to the T1 group. In contrast, T4 tumor stage did not appear to impact both metrics significantly more compared to the T1 stage. We suspect that the primary factor influencing these outcomes is tumor size, while the T4 group also includes patients with anaplastic and metastatic tumors, irrespective of tumor size [30].

There is currently no consensus regarding the impact between primary closure and flap-based reconstruction on speech outcomes, with mixed results reported in the literature [8, 45]. In our study, no significant differences could be discerned between primary closure and free flap reconstruction. However, primary closure was found to be significantly less impactful on speech intelligibility compared to bone flap reconstruction shortly after and up to 6 months post-treatment with intelligibility restoring towards the pre-treatment state after 12 months for both types. Notably, local flap reconstruction had a significantly more severe impact at 1 and 6 months post-treatment on both outcome metrics in comparison to all other reconstruction types. A possible explanation is the combined impact of tumor incision and local tissue removal in the oral cavity in local flap reconstruction, which is not present in other reconstruction types. We note that these results should be interpreted cautiously as the sample size of patients with local flap surgery is small in this study (*n* = 4). There was no statistically significant difference in both speech intelligibility and voice profile between primary closure and flap-based reconstruction 12 months post-treatment, indicating that reconstruction type mainly impacts speech and voice outcome only shortly after treatment.

It is known that radiotherapy during oncological treatment can potentially affect various tissues located in the oral cavity related to speech production [46]. Surgery with adjuvant radiotherapy is generally considered to negatively affect speech outcomes [14, 47, 48]. However, the impact of standalone radiotherapy compared to surgery is currently under-evaluated and no clear consensus has been reached on the most effective treatment for maintaining speech function [14]. In this study, the LMM analysis found no significant difference between surgical treatment and both standalone and adjuvant radiotherapy on speech intelligibility, suggesting other co-dependent variables are responsible for the observed degradation at the descriptive level. However, we observe a significant and substantial impact on voice profile at 6 and 12 months post-treatment for both radiotherapy groups. These outcomes suggest radiotherapy has a greater impact on voice profile compared to surgery without an additional reduction in speech intelligibility. The impact on voice profile may be attributed to radiation-induced toxicities, including oral mucositis, tissue fibrosis, and xerostomia [46].

To the best of our knowledge, this study is the first to employ speaker embeddings to assess changes in voice profile during oral oncological treatment. Other research related to objective measurements of voice profile are currently restricted to specific spectral properties but the relationship between individual spectral properties and the overall voice profile of the patient is currently unclear [41], limiting their usefulness in a clinical setting. While ASR-based intelligibility methods have produced satisfactory results for measuring objective speech outcomes [17], major disadvantages are still present: the method is text- and language-dependent, sensitive to acoustic recording conditions, and can be influenced by non-linguistic factors (e.g., fluency) [49]. In contrast, our proposed method does not require a reference transcription, is robust to recording conditions, and is language-independent, making it a compelling option to address the often expressed need of standardized methods to assess speech outcome after oral oncological treatment [14, 50].

Some limitations were present in the current study. First, while a recent study has demonstrated a strong correlation between speaker embedding similarity and human perceptual assessment of voice similarity in healthy speakers [22], no comparable data currently exists for patients with oral cancer. In addition, no studies are available describing the relationship between speaker embedding similarity and qualitative voice metrics commonly used in speech therapy (e.g., CAPE-V or GRBAS ratings), which could provide a more granular interpretation of the results in this study. Second, healthy individuals only had one measurement moment, resulting in the inability to establish the test–retest reliability of our objective metrics for voice profile and speech intelligibility in the control group. Third, the results of post-treatment measurement moments could be influenced due to the passing away or dropping out of patients in previous measurement moments, often individuals exhibiting a greater speech impairment.

Future research is needed to corroborate the results of this study, especially considering the strong association between speech outcomes following oral oncological treatment and the quality of life of patients [5, 6]. Our findings suggest that tumor stage and surgical reconstruction type significantly influence both voice profile and speech intelligibility post-treatment. Notably, the choice of primary closure versus flap-based reconstruction appeared to have mainly temporary effects on speech intelligibility, with no significant differences observed at the 12-month mark. The impact of radiotherapy, either solely or combined with surgery, was only significant on voice profile rather than intelligibility. Future studies should further examine the relationship between speaker embedding similarity and perceptual assessment of voice similarity by trained speech and language therapists, particularly in patients with oral cancer. Finally, further exploration is needed to assess the effectiveness of speaker embeddings as a diagnostic tool, potentially leading to more objective and automated methods for assessing voice profile in clinical settings.

## Conclusion

In this study, we found several demographic and clinical factors associated with objective measurements of voice profile and speech intelligibility after treatment of patients with oral cancer. Our findings advocate for further research of speaker embeddings as a robust and practical tool for evaluating voice profile after oral oncological treatment, aiming to improve the clinical assessment and treatment planning for these patients.

## Supplementary Information

Below is the link to the electronic supplementary material.

Supplementary File 1 (DOCX 204 KB)

## Data Availability

No datasets were generated or analysed during the current study.
