# Supplementary material for: Factors influencing objective voice profile and speech intelligibility of patients after oral oncological treatment: a prospective cohort study
Source: Support Care Cancer. 2025 Jul 31;33(8):731. doi: 10.1007/s00520-025-09793-z (PMC12310887; doi:10.1007/s00520-025-09793-z)

# Appendix A. Text 1: ’Loos alarm op het strand’

Loos alarm op het strand. Groot alarm afgelopen donderdag op het strand van Zandvoort. De kustwacht was gewaarschuwd dat er een persoon die zich in het water had begeven vermist was. Hij was tegelijk met nog twee andere personen in zee gegaan en nu waren die twee personen komen vertellen dat ze hem misten. Ze waren er zeker van dat hij niet teruggegaan was naar het strand. De reddingsbrigade van Zandvoort en Muiden werden daarom ingeschakeld om deze ene persoon te zoeken. Ook werd een helikopter ingezet. Een ziekenauto werd in gereedheid gebracht. Het alarm dat was geslagen bleek loos te zijn want er bleken niet drie maar twee personen in het water te zijn geweest. Tot grote paniek leidde het alarm onder de badgasten echter niet. De meesten bleven kalm en sloegen het een en ander met belangstelling gade.

# Appendix B. Text 2: ’De tafel wordt iedere dag gedekt’

De tafel wordt iedere dag gedekt. Die sokken passen niet bij die das. Anna noemde de namen op. Ik heb een pakket te koop. Lachen is gezellig. De lapjeskat ligt op de stoel. Rob gaat leren voor zijn toets. Jan was het gras aan het maaien.

# Appendix C. PTSS and LWER estimations of linear mixed-effects models

The estimated PTSS and LWER outcomes by the linear mixed-effects models in this paper are given below. The ’:’ delimiter indicates an interaction term:

Estimated PTSS = 5*.*23 *−* 1*.*66 *·* M1 *−* 1*.*64 *·* M6 *−* 1*.*65 *·* M12 *−* 0*.*01 *·* T2 + 0*.*22 *·* T3 + 0*.*18 *·* T4 *−* 0*.*05 *·* surgery&RT *−* 0*.*07 *·* RT + 0*.*09 *·* local_flap *−* 0*.*16 *·* free_flap *−* 0*.*15 *·* bone_flap *−* 0*.*32 *·* T2:M1 *−* 0*.*85 *·* T3:M1 *−* 0*.*41 *·* T4:M1 *−* 0*.*26 *·* T2:M6 *−* 1*.*21 *·* T3:M6 *−* 0*.*39 *·* T4:M6 *−* 0*.*17 *·* T2:M12 *−* 0*.*85 *·* T3:M12 *−* 0*.*27 *·* T4:M12 *−* 0*.*58 *·* surgery&RT:M1 *−* 0*.*53 *·* RT:M1 *−* 0*.*79 *·* surgery&RT:M6 *−* 0*.*82 *·* RT:M6 *−* 0*.*57 *·* surgery&RT:M12 *−* 0*.*95 *·* RT:M12 *−* 1*.*51 *·* local_flap:M1 *−* 0*.*27 *·* free_flap:M1 *−* 0*.*43 *·* bone_flap:M1 *−* 1*.*11 *·* local_flap:M6 *−* 0*.*01 *·* free_flap:M6 *−* 0*.*61 *·* bone_flap:M6 *−* 0*.*74 *·* local_flap:M12 *−* 0*.*13 *·* free_flap:M12 *−* 0*.*57 *·* bone_flap:M12

Estimated LWER = *−*1*.*97 + 0*.*01 *·* age + 0*.*19 *·* smoking *−* 0*.*01 *·* M1 *−* 0*.*07 *·* M6 *−* 0*.*12 *·* M12 *−*

0*.*08 *·* T2 *−* 0*.*06 *·* T3 *−* 0*.*04 *·* T4 *−* 0*.*02 *·* surgery&RT + 0*.*12 *·* RT *−* 0*.*19 *·* local_flap + 0*.*02 *·* free_flap + 0*.*02 *·* bone_flap + 0*.*12 *·* T2:M1 + 0*.*12 *·* T3:M1 + 0*.*04 *·* T4:M1 + 0*.*09 *·* T2:M6 + 0*.*18 *·* T3:M6 + 0*.*09 *·* T4:M6 + 0*.*18 *·* T2:M12 + 0*.*24 *·* T3:M12 + 0*.*15 *·* T4:M12 + 0*.*1 *·* surgery&RT:M1 + 0*.*01*·*RT:M1 + 0*.*13 *·* surgery&RT:M6 *−* 0*.*02 *·* RT:M6 + 0*.*12 *·* surgery&RT:M12 + 0*.*02*·*RT:M12 + 0*.*48 *·* local_flap:M1 + 0*.*09 *·* free_flap:M1 + 0*.*28 *·* bone_flap:M1 + 0*.*34 *·* local_flap:M6 *−* 0*.*01 *·* free_flap:M6 + 0*.*21 *·* bone_flap:M6 + 0*.*08 *·* local_flap:M12 *−* 0*.*1 *·* free_flap:M12 + 0*.*16 *·* bone_flap:M12

# Appendix D. Speech intelligibility (LWER) and voice profile (PTSS) correlation

**Fig. E.1** Figure depicting correlation between speech intelligibility (LWER) and voice profile (PTSS) of patients included in this study at the post-treatment measurement moments. The scatterplot illustrates individual data points, with the red line representing the fitted regression model and the shaded area indicating the 95% confidence interval. The Pearson correlation coefficient between both metrics is -0.41, indicating a moderate negative relationship. This suggests that as speech intelligibility decreases (higher LWER), the voice profile of patients tends to change relative to the pre-treatment measurement moment (lower PTSS).


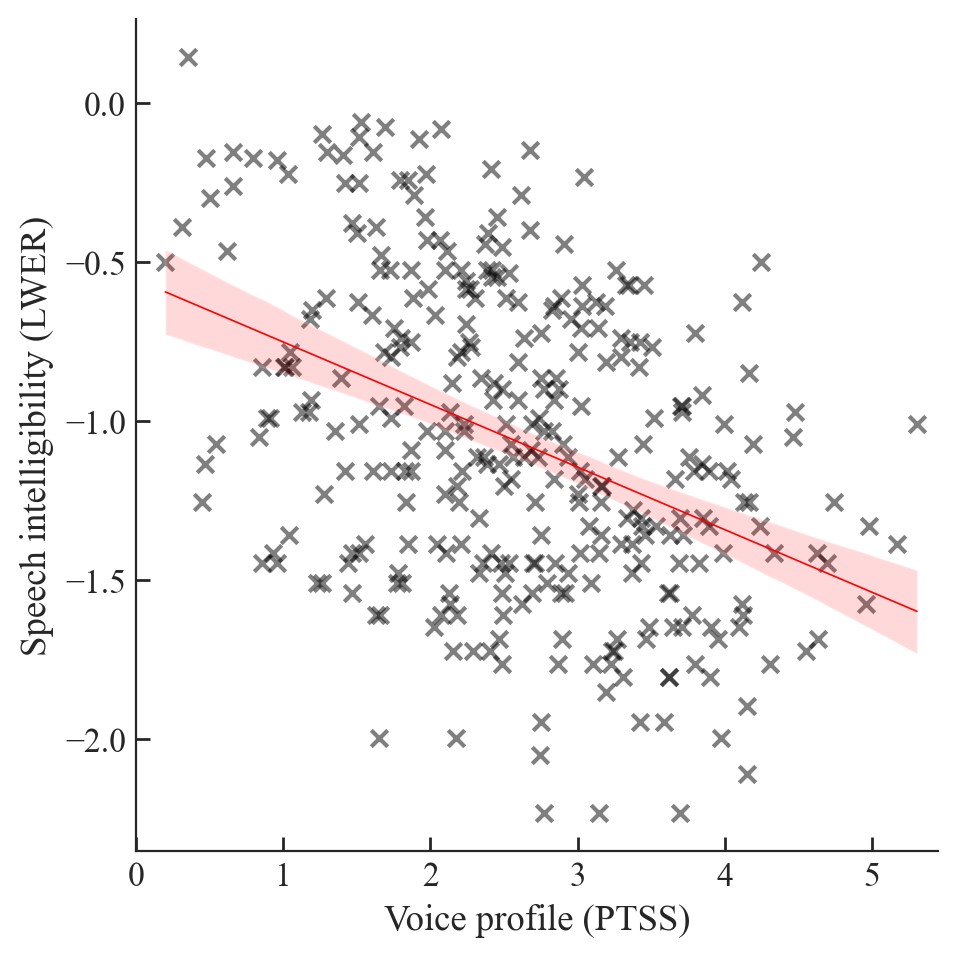

Supplement: Supplementary file 1 — (DOCX 204 KB) [file 520_2025_9793_MOESM1_ESM.docx]
